# Interventions to maintain essential services for maternal, newborn, child, and adolescent health during the COVID-19 pandemic: A scoping review of evidence from low- and middle-income countries

**DOI:** 10.7189/jogh.14.05024

**Published:** 2024-06-14

**Authors:** Diana Sagastume, Aloma Serra, Nina Gerlach, Anayda Portela, Lenka Beňová

**Affiliations:** 1 Institute of Tropical Medicine, Department of Public Health, Antwerp, Belgium; 2 London School of Economics and Political Science, Department of International Development, London, UK; 3 United Nations Development Programme, Department of Climate Change and Energy, Quito, Ecuador; 4 Independent consultant, Oldenburg, Germany; 5 World Health Organization, Department of Maternal, Newborn, Child and Adolescent Health and Ageing, Geneva, Switzerland

## Abstract

**Background:**

The coronavirus disease 2019 (COVID-19) pandemic had challenged health systems worldwide, including those in low- and middle-income countries (LMICs). Aside from measures to control the pandemic, efforts were made to continue the provision and use of essential services. At that time, information was not organised and readily available to guide country-level decision-making. This review aims to summarise evaluated interventions to maintain essential services for maternal, newborn, child, and adolescent health in response to COVID-19 in LMICs, in order to learn from the interventions and facilitate their use in the next disruption.

**Methods:**

We conducted a scoping review by Embase, MEDLINE, and Global Health for literature published between 1 January 2020 and 26 December 2022, without restrictions for language. We extracted information about the setting, population targeted, service type, intervention, and evaluation from the included studies and summarised it both quantitatively and narratively.

**Results:**

We retrieved 11 395 unique references and included 30 studies describing 32 evaluated interventions. Most interventions (84%) were implemented in 2020, with a median duration of five months (interquartile range (IQR) = 3–8), and were conducted in Africa (34%) or Southeast Asia (31%). Interventions focussed on maintaining services for maternal and newborn health (56%) or children and adolescents (56%) were most common. Interventions aimed to address problems related to access (94%), fear (31%), health workers shortage (25%), and vulnerability (22%). Types of interventions included telehealth (69%), protocols/guidelines to adapt care provision (56%), and health education (40%); a few entailed health worker training (16%). The described interventions were mostly led by the public (56%) or non-profit (34%) sectors. Methodologies of their evaluations were heterogeneous; the majority used quantitative methods, had a prospective research design, and used output- and outcome-based indicators.

**Conclusions:**

In this review, we identified an important and growing body of evidence of evaluated interventions to maintain essential services for maternal, newborn, child, and adolescent health during COVID-19 in LMICs. To improve preparedness and responsiveness for future disruptions, managers for decision-makers in LMICs could benefit from up-to-date inventories describing implemented interventions and evaluations to facilitate evidence-based implementation of strategies, as well as tools for conducting optimal quality operational and implementation research during disruptions (e.g. rapid ethical approvals, access to routine data).

In March 2020, the World Health Organization (WHO) declared the severe acute respiratory syndrome coronavirus 2 (SARS-CoV-2) a pandemic. The spread of the disease caused by the virus, most commonly known as coronavirus disease 2019 (COVID-19), strained health systems worldwide, including those in low- and middle-income countries (LMICs), where health systems had often been overburdened in the past due to outbreaks or other catastrophic events [1]. To halt the spread of the virus, countries established public health and social measures such as movement restrictions that limit access to health services including essential services [2]. Consequently, the provision and the use of care (supply and demand issues) were severely impacted, including maternal, newborn, child, and adolescent health (MNCAH) services [3]. The indirect effects of these impacts were especially severe in LMICs [4]. For example, the subsequent effect of the COVID-19 measures impacted the availability and uptake of MNCAH care service, thus exacerbating maternal and neonatal morbidity and mortality [1,5]. While some measures were temporary, ongoing disruptions still existed in 2021 in over 90% of countries that had responded to the Global Pulse Survey launched by the World Health Organization (WHO) to investigate the continuity of essential health services during the pandemic [6,7].

In early 2020, the WHO published a scoping review which sought to learn from the actions taken to maintaining essential services during past disruptive events, including the first wave of COVID-19 [8]. The final report identified 53 interventions during the initial phase of the COVID-19 pandemic (search date – December 2020) and reported a gap in knowledge regarding interventions in LMICs (only 13 interventions) and a general lack of evaluations of the interventions. To update the knowledge base and address the gaps, we aim to identify and summarise interventions to maintain the use and provision of essential health services for MNCAH in response to the COVID-19 pandemic in LMICs, including those that were evaluated as interventions.

## METHODS

This study was guided by the standard principles of scoping reviews described in Arksey and O’Malley’s Framework [9]. The methodology itself was based on the previous scoping review, with modifications to meet the study objective [8]. We reported our findings following the PRISMA-ScR guideline [10].

### Definitions

For purposes of this review, we defined target health areas as follows:

− Maternal health: the health of women during pregnancy, childbirth, and postnatal period, and reproductive health including only the following essential services: family planning, abortion care, and sexually transmitted infections.
− Newborn health: the health of babies from birth up to the first week of life.
− Child health: the health of children from one week to nine years.
− Adolescents health: the phase of life between childhood and adulthood, from 10 to 19 years.

Essential services for MNCAH included those contained within the WHO’s publication ‘Maintaining Essential Health Services: Operational Guidance for the COVID-19 Context’ [7] (**Box 1**).

Box 1 **Essential services for MNCAH included in this scoping review**

The following MNCAH services were included: essential prevention and treatment services for communicable diseases, including immunisations; services related to reproductive (family planning, abortion care, and sexually transmitted infections) and maternal health, including during pregnancy, childbirth, and postnatal period; core services for MNCAH; provision of medications, supplies, and support from health care workers for the ongoing management of chronic diseases, including mental health conditions; critical facility-based therapies; and management of emergency health conditions and common acute presentations that require time-sensitive intervention; and auxiliary services, such as basic diagnostic imaging, laboratory, and blood bank services. For ‘critical facility-based care,’ only newborn intensive medical care was included. We considered neonatal intensive care units (NICUs) for preterm and low birth weight babies to be essential services, so this information had to be stated in the title or abstract.

### Search strategy

We conducted a systematic literature search in MEDLINE via PubMed, Embase, and Global Health for studies published between 1 January 2020 and 26 December 2022 (date on which the search was carried out). We set no language restrictions. The search strategy involved a combination of the keywords ‘MNCA’ AND ‘health services’ AND ‘COVID-19’ AND ‘characteristics of COVID-19 (e.g. lockdown)’ AND ‘interventions’ AND ‘evaluation’ and their synonyms using Boolean operators (pages 3–4 in the **Online Supplementary Document**). We uploaded and managed the search results within Covidence. Two researchers (AS, NG) independently screened the titles and abstracts of the retrieved studies, followed by the full text of the ones remaining after the initial screening stage. Ten per cent of the studies were assessed in duplicate as a quality check. The researchers discussed and resolved any discrepancies by consensus or by consulting with a third researcher.

### Eligibility criteria

Prior to the title and abstract screening, the team developed a methods guide and established the inclusion and exclusion criteria. Studies published in an academic journal could be included if they detailed an intervention to maintain the use or provision of MNCAH essential services during COVID-19. The intervention itself had to address the target population directly or via health workers. The studies needed to provide an evaluation of the intervention, using qualitative, quantitative, or mixed methods approaches. For feasibility, and because of the gap identified by the previous report, we decided to only include studies conducted in LMICs, using the World Bank country income classification from 2022 [11].

We excluded literature reviews, conference abstracts, articles without a full text, or studies using ecological, theoretical, and simulation/modeling designs. Also, we excluded studies if they were published in a language not mastered by the investigators (e.g. Chinese). We also excluded studies addressing the management of COVID-19 or interventions addressing clinical outcomes (e.g. impact on labor) rather than the health service, as well as studies where the intervention focussed on medical students without targeting the provision of essential services (e.g. changes in the study curriculum). Lastly, we excluded interventions implemented through a specialised health service provider (e.g. oncologist, orthopedics, and psychiatry) or that included food provision services (e.g. provision of school meals).

### Data extraction

Three researchers (AS, NG, DS) independently extracted the information from the eligible studies in duplicate using an Excel template developed by the study team. If multiple publications reported on different stages of the same intervention, the different stages were considered as independent interventions and handled as such. Similarly, if a study reported on various interventions, we considered each intervention independently and extracted the data accordingly.

The extracted information included publication data, geographical setting, population targeted by the intervention, type of essential service, problem addressed by the intervention, intervention characteristics, and evaluation characteristics (page 5 in the **Online Supplementary Document**).

In addition to the approach detailed above and to facilitate synthesis, we used methodologies from the previous report [8] and classified the type of problem, the topic of the problem, and the type of intervention using pre-defined thematic categories, with multiple classification being possible. The aforementioned report developed a framework that summarised the main types of problems in maintaining MNCAH services provision and use in response to disruptive events [8]. We applied the framework in this study, allowing for the classification of the type of problem as ‘decrease in supply,’ ‘decrease in demand,’ ‘increase in demand,’ or a combination thereof (e.g. ‘increase in demand and decrease in supply’). ‘Decrease in supply’ indicated problems such as suspension or reduction of care provision, staff re-assignment to COVID-19 wards, lack of school-based care, or supply chain disruptions. ‘Decrease in demand’ referred to service users’ inability to reach facilities (e.g. due to lockdown measures) or populations’ unwillingness to use services (e.g. due to lack of trust). ‘Increase in demand’ included situations such as exacerbated or newly arising health needs, including those related to mental health, health education, preventive care, or an increase in poverty, unemployment, isolation, or vulnerability. We classified the problems into the following topics: access, fear, vulnerability, health worker shortage, delays in service provision, rumors/misconceptions, or aggravated health risks (page 5 in the **Online Supplementary Document**). Meanwhile, we categorized the types of interventions as telehealth, protocols/guidelines, health education, or health worker training.

### Synthesis of results

We described the eligible studies through evidence tables. Specifically, we summarised categorical variables using counts and percentages, and continuous variables using medians and interquartile ranges (IQRs). We conducted a visualisation and narrative synthesis by mapping the type of population, type of intervention, and method of evaluation to the type of problem. We summarised evaluations of the interventions by the type of outcomes (output-, outcome-, impact-based) and the metrics used to conduct the evaluations. We also identified and summarised recurrent themes related to the problems being addressed by the interventions, the implementation of the interventions, and the original authors’ reflections on the findings of the evaluations. The main purpose of this summary was not to identify the most effective interventions, but rather to synthesise lessons learned which could be helpful, primarily for policy-makers to guide decision-making in a future disruptive event, and secondarily, to inform evidence-synthesis and research priorities.

## RESULTS

**Figure 1:**
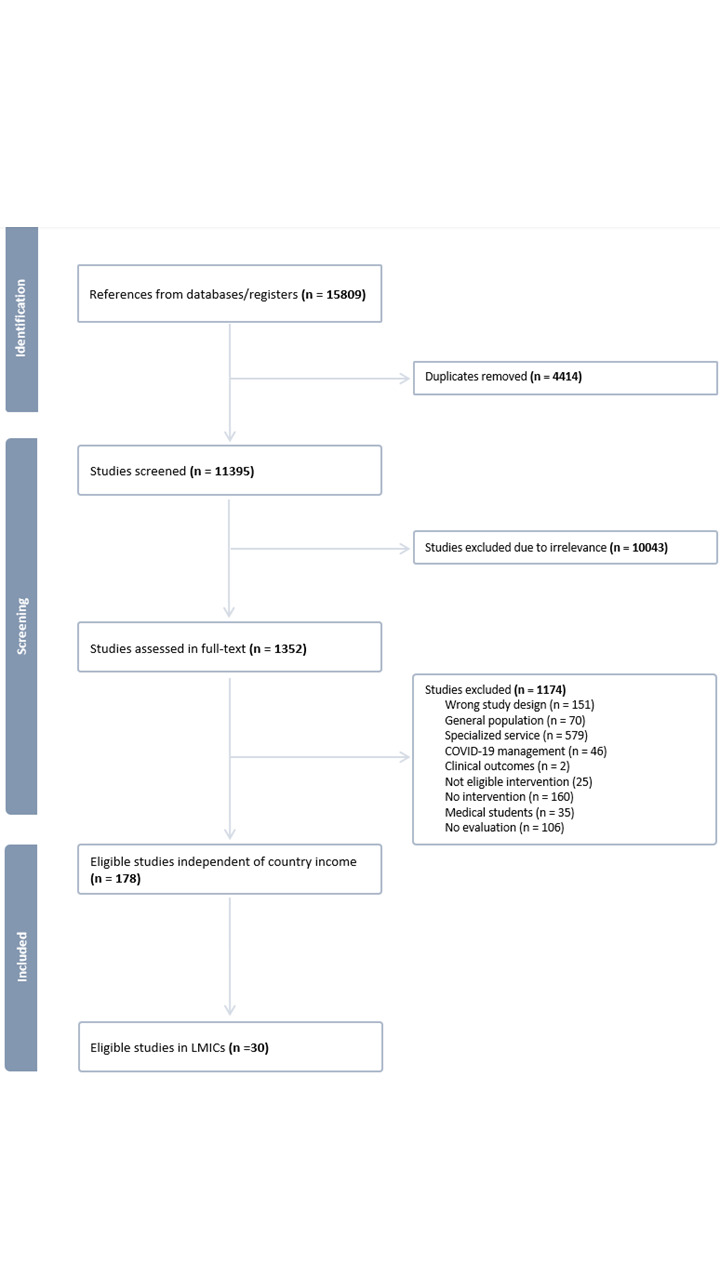
Flowchart – study selection process of eligible studies

We identified 15 809 records through searches in the three databases; 4414 were duplicates, with 11 395 unique references remaining following deduplication. After title and abstract screening, 10 034 references were considered irrelevant. The remaining 1352 references were reviewed in full text; 1174 were excluded, resulting in 178 studies being considered eligible. After this step, we included 30 studies from LMICs this review (**Figure 1**; Table S1 in the **Online Supplementary Document**).

The 30 included studies contained information about 32 unique interventions that had been evaluated (**Table 1**). Most were published in 2022 (59%). The most represented region was Africa (34%), with three studies being conducted in Zambia [12–14], three in Zimbabwe [15–17], two in Nigeria [15,18], and one in Cameroon [19], Kenya [15], and South Africa [20] each. Further, 31% of the interventions were carried out in Southeast Asia, including six in India [21-26], two in Indonesia [27,28], and one in Thailand [29] and Bangladesh [30] each. Five interventions were conducted in the Eastern Mediterranean (16%), and one each in Iran [31], Jordan [32], Lebanon [33], Pakistan [34], and Somalia [35]. Fewer interventions were conducted in the region of The Americas (9%), including two in Brazil [36,37] and one in Mexico [38], or in the Western Pacific region (6%), including two studies conducted in China [39,40], and only one study in Europe (3%), specifically in Turkey [41]. The interventions targeted different populations, mainly women (44%) and newborns (34%), followed by adolescents (28%), children (25%), and partners/parents (15%). About half of the interventions focussed on maintaining essential health services for maternal and newborn populations (56%) or children and adolescents (56%), and a slightly smaller number addressed reproductive health services (38%). Few interventions targeted vaccinations (13%) or mental health (6%). Of the 32 interventions, 59% targeted multiple populations and health services simultaneously.

**Table 1:** Summarised characteristics of the interventions described in included studies*

	Number of interventions (n = 32)†
**Publication year**	
2020	6 (18.8)
2021	7 (21.9)
2022	19 (59.4)
**Setting**	
WHO region‡	
*Africa*	11 (34.4)
*Southeast Asia*	10 (31.3)
*Eastern Mediterranean*	5 (15.6)
*The Americas*	3 (9.4)
*Western Pacific*	2 (6.3)
*Europe*	1 (3.1)
Area type	
*Urban and rural*	3 (9.4)
*Urban/peri-urban*	3 (9.4)
*Rural*	1 (3.1)
*Not specified*	25 (78.1)
**Population**	
Type§	
*Women*	14 (43.8)
*Newborns*	11 (34.4)
*Children*	8 (25.0)
*Adolescents*	9 (28.1)
*Partner/parents*	5 (15.6)
*Multiple*	22 (68.8)
**Health service**	
Type§	
*Mental health*	2 (6.3)
*Maternal and newborn*	18 (56.3)
*Child and adolescent*	18 (56.3)
*Reproductive health*	12 (37.5)
*Vaccination*	4 (12.5)
*Multiple*	19 (59.4)
**Problem**	
Type	
*Decrease demand*	2 (6.3)
*Decrease demand + decrease in supply*	13 (40.6)
*Decrease supply*	9 (28.1)
*Increase demand + decrease in supply*	6 (18.8)
*Increase demand*	2 (6.3)
Topic§	
*Access*	30 (93.8)
*Fear*	10 (31.3)
*Vulnerability*	7 (21.9)
*HCW shortage*	8 (25.0)
*Delays in service provision*	5 (15.6)
*Rumors/misconceptions*	5 (15.6)
*Aggravated health risk*	5 (15.6)
*Multiple*	24 (75.0)
**Intervention**	
Setting§	
*Hospital*	22 (68.8)
*Community*	12 (37.5)
*School*	0 (0.0)
*Multiple*	2 (6.3)
Implementation year	
*2020*	27 (84.4)
*2021*	3 (9.4)
*2020–21*	1 (3.1)
*Not specified*	1 (3.1)
Duration in months, median (IQR)‖	5 (3–8)
New or adapted intervention	
*New*	15 (46.9)
*Adaptation*	14 (43.8)
*Not possible to determine*	3 (9.4)
Sector of implementation§	
*Public sector*	18 (56.3)
*Private sector*	1 (3.1)
*Non-profit sector*	11 (34.4)
*Multiple*	2 (6.3)
Type§	
*Telehealth*	22 (68.8)
*Protocols/guidelines*	18 (56.3)
*Health education*	13 (40.6)
*HCW training*	5 (15.6)
*Multiple*	19 (59.4)
**Evaluation**	
Method	
*Qualitative*	4 (12.5)
*Quantitative*	18 (56.3)
*Mixed methods*	10 (31.3)
Approach	
*Prospective research design*	22 (68.8)
*Retrospective (routine) data*	8 (25.0)
*Both*	2 (6.2)

HCW – health care worker, IQR – interquartile range

*Presented as n (%) unless otherwise specified.

†The output of this table is based on 32 interventions derived from 30 studies.

‡The countries included in each WHO region are Cameroon (n = 1), Kenya (n = 1), Nigeria (n = 2), South Africa (n = 1), Zambia (n = 3), and Zimbabwe (n = 3) in Africa; Bangladesh (n = 1), India (n = 6), Indonesia (n = 2), and Thailand (n = 1) in Southeast Asia; Iran (n = 1), Jordan (n = 1), Lebanon (n = 1), Pakistan (n = 1) and Somalia (n = 1) in Eastern Mediterranean; Brazil (n = 2) and Mexico (n = 1) in The Americas; China (n = 2) in Western Pacific; and Turkey (n = 1) in Europe.

§Multiple categorisations possible.

‖The duration of the intervention is based on 25 interventions. For the interventions that were considered an adaptation, the duration was accounted for only after the adaptation.

The 32 interventions were classified by the type of problem(s) they addressed. Two types were addressed more frequently than single problems; ‘decreased demand and decrease in supply’ was identified in 41% of the interventions and ‘increase in demand and decrease in supply’ in 19%. Single problems were addressed in the remainder of the interventions: ‘decrease in supply’ (28%), ‘decreased demand’ (6%), and ‘increase in demand’ (6%). Almost all problems were related to the topics of ‘access’ (94%), followed by ‘fear’ (31%), ‘health workers shortage’ (25%), and ‘vulnerability’ (22%). Fewer interventions targeted problems categorised as ‘delays in service provision’ (16%), ‘rumors/misconceptions’ (16%), or ‘aggravated health risks’ (16%).

Most of the 32 interventions took place in hospitals (69%) or at the community level (38%); none were related to school-based health services. Among the 25 studies that specified the intervention duration, the median was 5 months (IQR = 3–8). Of all the 32 interventions, 47% were newly developed interventions to maintain essential services, while 44% could be characterised as adaptations of ongoing (pre-COVID-19) interventions. Half of the interventions were implemented by actors in the public sector (56%), followed by the nonprofit sector (35%), while a few were implemented by the private sector (3%) or by actors across multiple sectors (6%). About 69% of the interventions were telehealth-related, 56% implemented protocols/guidelines to adapt care provision, 40% included health education, and a few incorporated health worker training (16%). Three of the 32 interventions were governmental initiatives (led by the Ministries of Health of India and Zambia) and one intervention was implemented by a social security agency (the Mexican Institute of Social Security).

Regarding the evaluations of the included interventions, most used quantitative methods (56%), 31% used mixed methods, and four studies (13%) used qualitative methods only. The evaluations were conducted using different approaches: 69% had a prospective research design, 25% used retrospective (predominantly routinely collected health service) data, and two studies (6%) used both approaches.

**Figure 2:**
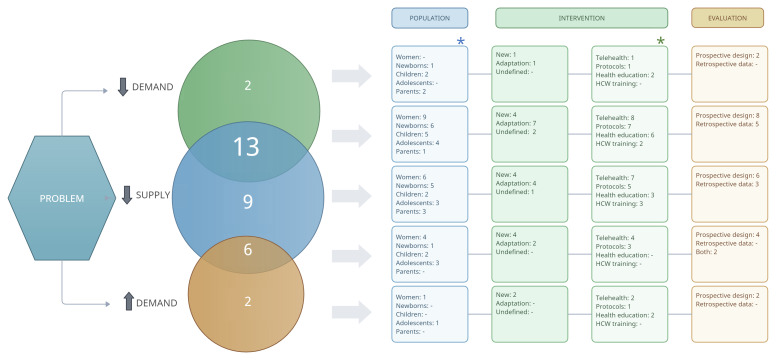
Mapping the type of population, type of intervention, and type of evaluation of the interventions by type of problem (32 interventions). *Multiple categorisation possible.

**Figure 2** shows that the interventions addressing problems related to a ‘decrease in demand’ frequently used health education as a type of intervention and tended to be evaluated using prospective research designs. Interventions aiming to address the ‘decrease in supply’ tended to use approaches such as telehealth and adaptations to protocols/guidelines to adapt care provision; the majority of them were also evaluated using prospective research design. The only clear pattern suggested from the comparison between the type of problem and whether the intervention was considered ‘new’ or ‘adaptation’ was that those interventions considered as an ‘adaptation’ aimed to tackle a ‘decrease in supply,’ whether or not the interventions included an ‘increase or decrease in demand.’ We observed no clear pattern when contrasting the type of population with the type of problem.

The type of outcomes and metrics used in the evaluation of the included interventions varied widely, with output- and outcome-based indicators being used most frequently. Output indicators included the number of referrals, number of people reached, number of appointments/consultations, and more specific ones like service coverage, number of deliveries, immunisation uptake, etc. Outcome-related indicators included knowledge, perceptions, attitudes, or satisfaction, particularly among intervention beneficiaries, their parents, or health workers. None of the studies reported impact metrics (e.g. mortality or morbidity indicators).

We identified three major recurrent themes. The first theme was that COVID-19 exacerbated pre-existing problems because MNCAH essential services were functioning sub-optimally before the pandemic. The second theme is linked to this exact type of pre-existing dysfunction, as it captured the need for and importance of contextual relevance of the interventions, particularly by engaging and collaborating with local stakeholders and communities. The third theme was specifically related to the predominance of telehealth as a type of intervention. Examples of telehealth interventions include teleconsultations, telemonitoring, and intervention delivery via texting (e.g. SMS or WhatsApp messaging), phone calls, or videoconferences; digital platforms for interaction and exchange of information (e.g. Facebook groups); and the development or adaptation of digital applications. Telehealth interventions were widely praised for reducing face-to-face contact in health facilities, saving financial resources for public facilities and service users, decreasing the risk of COVID-19 infection by avoiding personal contact, and helping alleviate anxiety symptoms. Conversely, telehealth-related interventions were also seen as exacerbating inequities in access to essential health services due to challenges related to equipment, connectivity, and literacy. This particularly affected rural households and lesser-educated populations. Also, concerns about privacy and data protection, longer waiting times, and payment difficulties were raised.

## DISCUSSION

This review provides an overview and synthesis of 30 studies describing 32 evaluated interventions to maintain the use and provision of MNCAH essential services during the COVID-19 pandemic in LMICs. This study outlines several examples of rapid problem identification, solution implementation, and evaluation during a period of uncertainty and health emergency, illustrating the feasibility of actions despite the difficult environment and restrictions for evaluations.

Our findings suggest that, during the first two years of the COVID-19 pandemic (2020–21), a ‘decrease in demand’ and/or ‘decrease in supply’ were the key obstacles to the use and provision of essential services for MNCAH in LMICs. These issues mostly arose as a result of public health and social measures that imposed restrictions on face-to-face consultations and physical access to health facilities in general [42]. Fear and health worker shortages were also reported as frequent sources of problems. These problems are interconnected and partially explained by the unknown situation experienced by the general population and health workers [43]; the reallocation of health workers to prioritise COVID-19 care; and the reduction of the health workforce (as COVID-19 also spread among this population) [44]. These problems highlight the difficulties in managing COVID-19 risks and care, as well as a lack of preparedness for a disruptive event in general, and in particular an epidemic caused by an infectious respiratory illness. In response to the identified problems, a variety of interventions were implemented, mainly led by actors in health facilities and research institutions in the public sector (e.g. public hospitals) or non-profit organisations. Although interventions targeted all MNCAH population groups, the majority focussed on maternal and newborn care. The characteristics of the interventions varied widely in terms of duration (ranging between 2 weeks to 16 months) and scale (from one hospital to nationwide). Also, interventions were classified as a mix of new interventions (e.g. changing face-to-face consultations to telehealth) and adaptions of ongoing (pre-COVID-19) interventions, whose implementation may have been accelerated to meet the demands of this period. The most frequently used types of interventions included telehealth, protocols, and health education. The evaluations of the interventions varied greatly, in the sense of methods, approaches, scales, and metrics, imposing difficulties to compare them. Most of the evaluations used quantitative methods, established a prospective research design (e.g. cross-sectional design, pre-post intervention), and used a combination of output- and outcome-based metrics. Yet, it is important to acknowledge that using available routine data might be more effective, easier, and time-efficient in crisis times than developing and implementing primary research. Moreover, the scale of evaluations ranged, for instance, from primary data from intervention beneficiaries to the use of routine data from a social security system.

Reflecting on the summary of the lessons learned on recurrent themes, several of the included studies highlighted the way the COVID-19 pandemic exacerbated existing health problems due to issues related to socioeconomic barriers such as the fragility of given contexts (e.g. a refugee camp [30]), socio-economic barriers to seeking care [20], or due to unavailability of health services for certain populations (e.g. adolescent girls [14]). Conversely, this also shows that the disruptions generated by the pandemic opened up new avenues for change. Moreover, the need for the contextualisation of interventions and engagement of local partners becomes particularly important when dealing with misinformation, like with COVID-19, as finding ways to connect with the audience depends on the context. Not all interventions can be directly translated to other settings. While we identified both positive and negative effects of adaptations using telehealth for maintaining MNCAH essential services, it still seems to be a promising tool that could be leveraged during future pandemics. In general, but particularly for telehealth interventions, further attention is necessary to address equity-related issues, namely those related to access to care for the most vulnerable population (e.g. those living in remote areas or people living in extreme poverty). Telehealth interventions should critically consider socio-demographic and economic disparities prior to implementation to avoid potential exacerbation of existing barriers to access to care. In this line, published evidence [45–47], has also stressed the importance of equity when moving forward with telehealth in LMICs[45,48] and the need for joint partnerships to exchange competencies and knowledge of telehealth infrastructures [45]. Moreover, challenges faced during implementation, predominantly reported for telehealth-related intervention, include higher costs due to equipment investment (e.g. laptops), connectivity (e.g. poor internet connection), need for extra training (e.g. training health workers to use new platforms), waiting times, online payment difficulties, language and communication barriers, among others. Another lesson learned identified by these findings is to ‘consider the use of already available resources.’ Some of the interventions proved strikingly straightforward, for example, by leveraging tools that health workers and service users employ in their daily lives (i.e. WhatsApp or Facebook groups) as a channel for implementing the intervention.

Summing up, our findings add to the body of existing evidence and joint initiatives on actions to maintain MNCAH essential services in response to COVID-19 in LMICs [8,49-51]. We extend the literature by incorporating a synthesis of 32 interventions that shed light on problem identification, solution implementation, and evaluation during the COVID-19 pandemic in LMICs, hence these findings can contribute to the preparedness for future pandemics or health crises.

We also identified gaps in the included studies from several perspectives. The identified interventions were not equally implemented or reported across geographical regions, with only a few interventions being conducted in the Americas and Western Pacific regions. Moreover, the included studies rarely provided a rich description of the context within which the implementation happened. The acceptability of the intervention beneficiaries and providers was likewise either poorly measured or reported, except for some telehealth interventions that measured satisfaction. This limits, for example, the generalisability of overall findings or the identification of factors enabling the implementation of a specific type of intervention or evaluation. Also, the majority of the interventions were of short duration and offered no information related to post-intervention effects or long(er)-term follow-up. This information is key to providing insights into the effectiveness and sustainability of the long-term effect of the interventions and the scalability of the intervention to other populations, hence providing the potential for these interventions to be integrated into existing health systems infrastructures. Furthermore, the evaluated interventions were less focussed on child and adolescent health. This aligns with the fact that none of the interventions were delivered through schools, perhaps due to their closure during the pandemic. Nevertheless, the question remains whether the services that remained open were able to compensate for the essential services potentially provided in school for children and adolescents [8]. Also, a few of the evaluated interventions were aimed at maintaining the provision of mental health and vaccination services, which are of major importance for preventive health during a pandemic.

Regarding the interventions, we found no interventions from 2022, although our search was conducted in December 2022. This indicates that the included studies belong to the most critical period of the COVID-19 pandemic. A search update is necessary to understand the interventions delivered during the different phases of COVID-19. Lastly, governmental-led, particularly large-scale/national interventions were largely absent in this evidence synthesis, independently of the efforts reported previously [6]. This raises the question of whether the dearth of broader-based national/regional strategies in this study is due to a lack of interventions, evaluations, effect of interventions, and/or publication of such efforts. Nevertheless, it is important to note that, although the previous WHO report considered interventions conducted in high-income countries, the identified interventions to maintain services for MNCAH in response to early stages of COVID-19 tended to be individual-based strategies (e.g. initiatives led by a single general practitioner) [8]. Contrastingly, in this synthesis, which included studies conducted in a later stage of COVID-19, interventions differed in scale, from those delivered within one or several health facilities or non-profit organisations to a few of those by national health authorities.

Several multilateral institutions, including the World Bank's Pandemic Fund [52] or the Pandemic Accord by the WHO [53], are looking to strengthen pandemic prevention, preparedness, and response, alongside countries’ plans to build capacities for more resilient health systems. Such plans should consider promoting mechanisms to ensure that lessons can be learned from such interventions using rigorous research methods and data. The studies identified in this review show that, even in low-resource settings facing sub-optimally functioning health systems and major societal disruptions, local stakeholders can accomplish maintaining essential services for MNCAH with context-adapted approaches. The results of such studies can then be aggregated in an up-to-date repository (inventory) of tested interventions and lessons learned to facilitate rapid implementation of effective context-appropriate actions that would facilitate cross-country learning. In addition, looking closer into those interventions that attempted recovery from the COVID-19 adaptations to pre-pandemic modalities – evidence not captured by our study – could bring additional insights on how to plan for and recover from disruptive events. Guidance for conducting good quality operational, implementation, and reporting research, including detailed descriptions of methodologies for implementation and evaluation, during disruptive situations is necessary; also highlighted in the previous WHO document [8]. Such recommendations would encourage and support better decision-making. Moreover, preparedness and response strategies for future pandemics could benefit from closer cooperation and exchange of best practices between single health facilities, and sub-national and national-level authorities.

This study has some limitations, a major one being the structural capacity to conduct and report research during a crisis period in constrained settings. It is possible that, in settings with higher levels of preparedness and resources (e.g. expertise, human, financial), implementers and researchers were more likely to publish their research during this period than those in settings that lacked such capacity. This publication bias means that valuable lessons on interventions and their evaluations might not be available in the published literature and were therefore not included in this review. Corollary, although the search was conducted in December 2022, this review only identified interventions implemented during the initial phase of COVID-19, between 2020 and 2021, therefore, interventions focusing on recovering from COVID-19 interventions are lacking. Moreover, eligible studies were potentially overlooked due to the absence of hand-searching approaches and grey literature searches, and the exclusion of a few studies published in languages not mastered by the researchers. Owing to the large heterogeneity across reported outcomes, we were unable to coherently summarise the impact of the interventions on MNCAH-related outcomes. We also acknowledge that the initial scope of this study was not limited to LMICs; this decision was taken to manage the scope of the review and to provide a clear-cut synthesis for those settings most affected by COVID-19.

## CONCLUSIONS

This scoping review summarised the published evidence on interventions to maintain essential services for MNCAH during the first two years of the COVID-19 pandemic in LMICs. It documented 32 examples of successful rapid problem identification, solution implementation, and evaluation across six WHO world regions during a period of vulnerability and health emergency. Our synthesis contains predominantly small-scale initiatives attempting to address locally specific issues related to access to health services by exploring the use of telehealth, establishing new or adapted protocols, or providing health education. The evaluations used mainly quantitative methods, established a prospective research design, and included output- and outcome-based metrics. Although the evidence was highly heterogeneous in scope and methods, key lessons can be summarised as the importance of contextualising interventions to local settings and prioritising the use of available resources. To strengthen preparedness and response of health systems to disruptions – including future pandemics – we encourage further investment in promoting mechanisms for up-to-date repository and evidence synthesis of implemented interventions and evaluations, including lessons learned, with the aim of facilitating learning, selection, and implementation of contextually-relevant and effective strategies across counties. We also recommend facilitating preparation for rigorous operational and implementation research on maintaining essential health services during such disruptions (e.g. rapid ethical approvals, access to high-quality routine data).

## Additional material


Online Supplementary Document
