# Supplementary material for: Interventions to maintain essential services for maternal, newborn, child, and adolescent health during the COVID-19 pandemic: A scoping review of evidence from low- and middle-income countries
Source: J Glob Health. 2024 Jun 14;14:05024. doi: 10.7189/jogh.14.05024 (PMC11170233; doi:10.7189/jogh.14.05024)
Supplement: Online Supplementary Document [file jogh-14-05024-s001.pdf]

Supplementary file - Interventions to maintain essential services for maternal, newborns, child, and adolescent health during the COVID-19 pandemic: A scoping review of evidence from low- and middle-income countries

Diana Sagastume[1], Aloma Serra [2,3], Nina Gerlach [4], Anayda Portela[5]*, Lenka Beňová[1]

Affiliations

[1] Institute of Tropical Medicine, Department of Public Health, Antwerp, Belgium

[2] London School of Economics and Political Science, Department of International Development, London, UK

[3] United Nations Development Programme, Department of Climate Change and Energy, Quito, Ecuador

[4] Independent consultant, Oldenburg, Germany

[5] World Health Organization, Department of Maternal, Newborn, Child and Adolescent Health and Ageing, Geneva, Switzerland

Table of content

Section 1: Methods 3
1.1 Search strategy 3
1.2 Data extraction specifications 5
1.3 Categorization of the problem’s topics 5
Section 2: Results 6
Table S1. Evidence table of included studies (n=30) 6
References 20

Section 1: Methods

1.1 Search strategy

Search algorithm:

- population AND health services AND circumstances AND specific terms for COVID-19 AND specific terms for interventions AND specific terms for evaluations

Search terms:

<u>Population</u>

Maternal OR antenatal OR ante-natal OR prenatal OR pre-natal OR gestati* OR childbirth OR birth OR intrapartum OR labo?r OR pregnancy OR vertical OR postpartum OR postpartum OR puerper* OR perinatal OR maternity OR reproductive or eclampsia OR preeclampsia OR fertil* OR fundal OR f?tal OR Newborn OR new-born OR new born OR postnatal OR post-natal OR neonatal OR kangaroo OR low birthweight OR low birth-weight OR pre?term OR prematur* OR post?term OR skin-to-skin OR breastfeeding OR breast-feeding OR lactat* OR Child* OR childhood OR infant OR postneonatal OR under five OR under-five OR underfive OR under 5 OR growth OR Adolescent* OR teen* OR youth* OR young adult* OR school-age OR young mother* OR young person* OR young people OR young wom?n OR Vulnerable population*

<u>Health services</u>

(Health adj3 delivery) OR (health adj3 utili?ation) OR (health adj3 services) OR (care adj3 provision) OR health-care OR healthcare OR (care adj3 worker*) OR (community adj3 worker*) OR obstetrician* OR midwife OR midwives OR nurse* OR (medical adj2 officer*) OR (clinical adj2 officer*) OR Health system* OR health facilit* OR maternity OR referral OR facility-based OR home-based OR school-based OR community-based OR Provision OR essential services OR essential healthcare OR indirect effect* OR collateral damage OR maintain* OR resilien* OR strengthen* OR sustain* OR mitigation strateg* OR cash transfer* OR user fee* OR helpline* OR digital OR telemedicine OR tele-health OR routine service* OR obstetric* OR family planning OR contraception OR abortion OR miscarriage OR postabortion OR post-abortion OR birth attendant OR skilled birth OR c?esarean OR c?section OR formula-feeding OR bottle-feeding OR breastmilk substitute OR p?ediatric* OR infant feeding OR infant nutrition

<u>Circumstances</u>

COVID OR Covid-19 OR SARS-CoV-2 OR coronavirus

<u>Specific terms for COVID-19</u>

Mental health OR Disrupt* OR PPE or Protect* OR IPC OR transport OR transportation OR access OR lockdown OR quarantine OR isolation OR self-isolation OR confinement

<u>Specific terms for interventions</u>

Intervention* OR polic* OR program* OR project* OR initiative* OR implement* OR manag* or monitor* OR activit*

Search strategy conducted in Ovid

# ▼	Searches	Results	Type	Actions
6	limit 5 to human [Limit not valid in Global Health; records were retained]	15809	Advanced	Display Results | More
5	limit 4 to yr="2020 -Current"	17512	Advanced	Display Results | More
4	1 and 2 and 3	17633	Advanced	Display Results | More
3	(evalua* or method* or assess* or apprais* or measur* or analy* or impact*).mp. [mp=ti, ab, hw, tn, ot, dm, mf, dv, kf, fx, dq, cw, bt, nm, ox, px, rx, ui, sy]	44788999	Advanced	Display Results | More
2	(intervention* or polic* or program* or project* or initiative* or implement* or manag* or monitor* or activit*).mp. [mp=ti, ab, hw, tn, ot, dm, mf, dv, kf, fx, dq, cw, bt, nm, ox, px, rx, ui, sy]	22632018	Advanced	Display Results | More
1	((Maternal or antenatal or ante-natal or prenatal or pre-natal or gestati* or childbirth or birth or intrapartum or labo?r or pregnancy or vertical or postpartum or post-partum or puerper* or perinatal or maternity or reproductive or eclampsia or preeclampsia or fertil* or fundal or f?tal or Newborn or new-born or new born or postnatal or post-natal or neonatal or kangaroo or low birthweight or low birth-weight or pre?term or prematur* or post?term or skin-to-skin or breastfeeding or breast-feeding or lactat* or Child* or childhood or infant or postneonatal or under five or under-five or underfive or under 5 or growth or Adolescent* or teen* or youth* or young adult* or school-age or young mother* or young person* or young people or young wom?n or Vulnerable population*) and ((Health adj3 delivery) or (health adj3 utili?ation) or (health adj3 services) or (care adj3 provision) or health-care or healthcare or (care adj3 worker*) or (community adj3 worker*) or obstetrician* or midwife or midwives or nurse* or clinician* or (medical adj2 officer*) or (clinical adj2 officer*) or Health system* or health facilit* or maternity or referral or facility-based or home-based or school-based or community-based or Provision or essential services or essential healthcare or indirect effect* or collateral damage or maintain* or resilien* or strengthen* or sustain* or mitigation strateg* or cash transfer* or user fee* or helpline* or digital or telemedicine or tele-health or routine service* or obstetric* or family planning or contraception or abortion or miscarriage or postabortion or post-abortion or birth attendant or skilled birth or c?esarean or c?section or formula-feeding or bottle-feeding or breastmilk substitute or p?ediatric* or infant feeding or infant nutrition) and (COVID or COVID-19 or SARS-CoV-2 or coronavirus) and (Mental health or Disrupt* or PPE or Protect* or IPC or transport or transportation or access or lockdown or quarantine or isolation or self-isolation or confinement)).mp. [mp=ti, ab, hw, tn, ot, dm, mf, dv, kf, fx, dq, cw, bt, nm, ox, px, rx, ui, sy]	33949	Advanced	Display Results | More

1.2 Data extraction specifications

The information extracted included

A. Publication data: author, year, and name of the academic journal
B. Geographical setting: country, WHO world region, and area type (rural, urban, both)
C. Population targeted by the intervention: MNCA population for whom the health service was provided and others (e.g., carers, partners/spouses of pregnant women, parents of newborns, children, and adolescents) if applicable, and their age range.
D. Essential services: maternal and newborn, child and adolescent, reproductive health, mental health (provided by the essential services), and vaccinations.
E. Problem addressed by the intervention.
F. Intervention characteristics: date of implementation, duration, if newly developed intervention or adaptation of ongoing intervention, scale (e.g., regional, one hospital), sector of intervention implementers (private, public, non-profit sector), and intervention's description.
G. Evaluation: method (quantitative, qualitative, mixed methods), approach (prospective research design, or retrospective data use including routine data), scale of evaluation (e.g., number of participants included in the evaluation, routine data related to a health facility), variables assessed in the evaluation, and the main results

1.3 Categorization of the problem's topics

Problems were classified into the following topics:

- Access: physical impossibility or difficulty of accessing the place where the health service is delivered
- Fear: fear of contracting COVID-19 leading, for example, to people not wanting to leave their homes
- Vulnerability: aggravated socio-economic situation as a result of COVID-19
- Healthcare workers shortage: lack of health professionals to deliver the services
- Delays in service provision: a situation in which the service has not stopped but has been severely disrupted causing postponement.
- Rumors/misconceptions: about the virus itself, the transmission, the symptoms, the cure, and its relationship to other diseases/conditions
- Aggravated health risks: clearly defined, aggravated health risk for an identified disease or condition that is directly worsened by COVID-19.

Section 2: Results

Table S1. Evidence table of included studies (n=30)

Author, year	Country	Health service	Problems identified related to COVID-19	Scale of the intervention	Description of the intervention	Duration of intervention	Evaluation methods	Scale of evaluation	Metric of evaluation
Ahmad, 2021 [1]	Lebanon	- Maternal and Newborn<br>- Child and Adolescent	- Movement restrictions<br>- Reduced access to infant and young child feeding (IYCF) support.<br>- Misconceptions and myths regarding breastfeeding and COVID-19.<br>- Reduced face-to-face affected women's perceptions, experiences, and practices of breastfeeding.	All governorates	Volunteer educators and lactation specialists were mobilized to scale up awareness-raising activities. These were also adapted to cover IYCF and COVID-19 (i.e., key messages to mitigate the misconceptions about IYCF in this context). They also expanded their one-to-one and group counseling to reach more people. This was done both remotely and in person with infection prevention control measures in place. Furthermore, a national hotline to report violations of the Breast Milk Substitute was established.	11 months	Quantitative	Data from all governates	Number of people receiving counseling support, people reached by awareness campaign
Ahmed, 2021 [2]	Somalia	- General health<br>- Maternal and Newborn	- Caregivers did not visit the health facilities as frequently as normal due to the fear of COVID-19<br>- Rumors, misconceptions, and stigma related to COVID-19, and its transmission (e.g., through breastfeeding).	20 health and nutrition facilities	Adaptations to a service of counseling sessions for mothers/caregivers of children 6-24 months of age and pregnant and lactating women for community-based management of acute malnutrition services, vaccination, antenatal care, postnatal care, or any other health services. Intervention included: key information messages in the local language in health facilities, the training of health workers on infant and young child feeding (IYCF) in the COVID-19 context, IPC measures implemented in individual and group counseling sessions, a reduced number of participants at group counseling sessions and an increase in the number of sessions held and IYCF counselors per health facility to support this, an increased number of handwashing stations at health facilities and the inclusion of people recovered from COVID-19 into	Not possible to determine	Quantitative	Data from the 20 health and nutrition facilities	Number of participants reached with counselling sessions.

					counselling sessions to help to address the stigma and rumors associated with the virus in the community.				
Barua, 2022 [3]	Bangladesh	- Maternal and Newborn<br>- Reproductive health	- Lack of access to transport for emergency obstetric services (before covid and exacerbated by the pandemic).	Several refugee camps	Implementation of a community-based referral project. This intervention addresses the second delay associated with seeking emergency obstetric and neonatal care in humanitarian settings by providing free transportation with ambulatory facilities and offering a robust referral mechanism.	8 months	Mixed Methods	100 intervention beneficiaries	Utilization of the referral hub as number of referrals and perception of service measured as the number of women saying they would recommend the service.
Devi, 2022 [4]	India	- Maternal and Newborn	- Difficulties faced by pregnant women in seeking appropriate antenatal care due to the imposed restrictions.	1 tertiary care hospital	Telehealth consultations were implemented to deliver antenatal care.	4 months	Quantitative	35 intervention beneficiaries	Awareness of teleconsultation and its outcome, patient's perceived satisfaction, and factors affecting the teleconsultation services.
Doubova, 2022 [5]	Mexico	- Maternal and Newborn<br>- Child and Adolescent<br>- Reproductive health	- The resource redistribution towards COVID-19 care and the strategies to reduce congestion in health facilities resulted in a decline in essential health services provision.	1523 primary health care facilities and 283 hospitals across 32 states	The Mexican Institute of Social Security (IMSS) launched a recovery policy to address the decline of services offered: 1) reconversion of previously repurposed COVID-19 hospitals back to routine care, 2) strengthening COVID-19 preventive measures, 3) adjusting essential health services governance, optimizing service delivery and organizing weekend health services, 4) implementation of telemedicine services, including virtual consultations for individuals with controlled chronic diseases in select clinics, 5) strengthening of preventive services and health promotion activities, and 6) essential health services monitoring.	8 months	Quantitative	Routine data from the IMSS including 1523 primary health care facilities and 283 hospitals across 32 states	The number of consultations for children under five with diarrhea, pneumonia, or malnutrition in primary care clinics, the total number of children who completed the final required dose of several vaccines, the number of reproductive-age women who used contraceptive services, number of antenatal care consultations, number of facility deliveries, and the number of caesarean section deliveries.
Enyama, 2020 [6]	Cameroon	- Child and Adolescent	- Adapt health service due to COVID-19 restrictions.<br>- Fear of pediatricians	Not specified	As a response to the drop in the number of visits to health facilities with a decrease in consultations and hospitalizations, many doctors have started providing telehealth. The consultations and advice were usually given through WhatsApp, normal phone calls, or through	Not possible to determine.	Quantitative	101 intervention implementers	Frequency of use of telehealth and use of protective equipment.

			getting infected and infecting their families.<br>- Parents' fear of being infected when leaving the house to visit a health facility.<br>- Lack of equipment (i.e., PCR).		Skype. There is also a secondary intervention related to protective equipment.				
Gadappa, 2022 [7]	India	- Maternal and Newborn	- COVID-19 made it more difficult to have a birth companion of choice as a result of the restrictions	1 large referral hospital	A policy was designed and implemented to increase the percentage of deliveries accompanied by birth companions. The policy has the following points: (1) inform women about what a birth companion is during antenatal care and to identify and register beforehand, (2) birth companions are informed about roles and responsibilities during labor and birth (3) antenatal education classes to prepare both the woman and birth companion for labor and birth journey - facilitated by a multidisciplinary team and comprising a manual and toolkit with information such as nonpharmacologic pain relief techniques. Special care and extra efforts were taken to implement this policy during COVID-19.	8 months	Quantitative	10387 intervention beneficiaries were measured as total deliveries.	Number of deliveries with birth companions, number of complications, cesarean section rate, and operative vaginal deliveries
Goyal, 2022 [8]	India	- Maternal and Newborn	- Difficulties faced by pregnant women in seeking appropriate antenatal care due to imposed restrictions.<br>- Difficulties encountered during delivery and postpartum period.	3 districts	The Health Ministry rolled out the 'eSanjeevani OPD' platform enabling patient-to-doctor telemedicine intervention.	Not possible to determine	Quantitative	1374 intervention beneficiaries and non-beneficiaries.	Accessibility to smartphone, if teleconsultation taken, mode of teleconsultation, teleconsultation experience, telemedicine as compared with an ordinary visit.

Hensen, 2022 [9]	Zambia	- Child and Adolescent<br>- Reproductive health	- Restrictions forced the hubs distributing Menstrual hygiene products (MHP) to close.<br>- Adolescent girls and young women (AGYW) have limited information about menstruation and access to MHP.	10 geographical areas within two peri-urban communities	Several adaptations were made to limit the number of AYP attending the hubs at one time. Mandatory mask-wearing and hand-washing before entering the hubs, and implementing an appointment system.	7 months	Mixed Methods	Data routinely collected by the programme including the 10 areas within two peri-urban communities	Uptake of service MHP and use of PPC points to redeem a reward MHP through: number of visits to the service, number of visits where pads were collected, total and hub-level % of visits where pads were collected, number of visits for rewards, number of reward visits where MHP collected, and the total and hub-level % of reward visits where MHP collected.
Joshi, 2022 [10]	India	- Child and Adolescent	- National lockdown disrupted the process to improve the quality of pediatric ward-round documentation by application of subjective, objective, assessment and planning (SOAP) format.	1 hospital	The intervention focused on writing patients' notes under the SOAP format during the 24 hours of a patient's stay. The general intervention included verbal reminders to use the SOAP format, individual oral feedback, and SOAP acronym display. Adaptions due to COVID-19 included particularly visual prompter on team compliance with SOAP and other activities were discontinued.	6 months	Qualitative	Data and feedback collected in 1 hospital	Median compliance with the SOAP format regarding the pediatric case files.

Khader, 2022 [11]	Jordan	- General health service,<br>- Child and Adolescent<br>- Vaccination	- Curfew suspended mass preventive vaccination campaigns and prevented caregivers from visiting health facilities for vaccinations.<br>- The vaccination rate for refugees was already low before COVID-19 and was potentially exacerbated by the lockdowns.	1 refugee camp	The app CIMA was initially launched in 2019 to facilitate the registration of children vaccination. During COVID-19, two volunteers in a refugee camp visited parents to help them install the app and explain the content of it. The staff explained the content of the app in terms of vaccination schedule, health promotion materials for vaccination, and parenting skills to their caregivers. They implemented an intervention study to support parents and caregivers in a refugee camp during the COVID-19 pandemic using a leaflet on caregiving under Covid-19, which was delivered using the smartphone application CIMA.	2 months	Mixed methods	1100 intervention beneficiaries + 4 intervention implementers	Registration of children and inquiries about children's vaccination status
Li, 2021 [12]	China	- Mental health<br>- Child and Adolescent	- Adolescents suffer a high level of emotional and behavioral problems, such as anxiety and depression as a result of the pandemic.	4 communities	1) Solution-focused brief therapy-based group intervention: an intervention group communicating primarily through mobile phone messages or calls was established. Members of the intervention group received the questions of solution-focused brief therapy in the form of mobile phone messages. The members sent their answers to the research team in the form of mobile phone messages. The instructors gave short and positive feedback to the members' answers, including positive guidance.<br>2) A short video health education related to the pandemic: It included routine health knowledge education given through video playing targeting physical and mental development characteristics and knowledge reserve level of 7 to 12-year-old students and the pandemic.	1 month	Quantitative	118 intervention beneficiaries	Self-rating Anxiety Scale (SAS) and Positive and Negative Affect Scale

Li, 2021 [13]	China	- Maternal and Newborn	- Disruptions in routine face-to-face pre-term infant follow-up	Neonatal intensive care unit from 1 hospital	Online follow-up was conducted by the same doctors who conducted face-to-face follow-ups with preterm infants before COVID-19. Doctors evaluated the babies' development status, feeding, excretion, nutrient supplementation, and vaccination through telemedicine visits and then provided corresponding guidance. Neurodevelopmental assessment and early development promotion guidance were implemented through the WeChat video. Both online and face-to-face follow-up included growth monitoring and evaluation, feeding consultation and guidance, nutrition supplementation, nursing and disease prevention guidance, and early development promotion guidance. For physical examinations, neuropsychological, and behavioral development monitoring, and evaluation, which cannot be completed online, doctors asked parents to describe the observation of the baby through daily life and demonstrate whether the baby could do some milestone activities via the WeChat video call.	3 months	Quantitative	79 intervention beneficiaries (babies) + 62 caregivers of the intervention beneficiaries.	The anxiety level of the mothers and families of pre-term infants, parents' satisfaction with online follow-up and level of agreement with the statement 'online follow-up answered all my questions' and 'online follow-up could replace face-to-face follow-up', follow-up rate between online and face-to-face follow-up of the preterm infants within 3 weeks after discharge.
Mackworth-Young, 2022 [14]	Zimbabwe	- Child and Adolescent<br>- Reproductive health	- Sexual and reproductive health (SRH) was impacted by the restrictions and the already under-resourced healthcare systems.<br>- The restricted access to SRH services threatens youth.	4 sites within three provinces	CHIEDZA is a community-based intervention to improve HIV outcomes in youth (16–24 years) that focuses on 1) access: community-based youth-friendly, 2) uptake and acceptability: service branding, confidentiality, and social activities, and 3) Content and quality: integrated HIV care cascade, high-quality products, and trained providers. During the lockdown, the intervention closed and reopened later as an essential service with several adaptations in place. Some examples include all services being moved from inside the community centers to outside, health booth tents being spaced apart, and having one wall open for ventilation. To preserve confidentiality, where feasible, the open wall faced a building or was positioned not to be visible to persons outside the tents. Masks were worn by all providers and clients, table surfaces were wiped after use, and handwashing facilities were offered. Social distancing rules were applied. The hours of service provision were limited. The package of health services offered remained the same.	5 months (estimated)	Qualitative	22 intervention implementers + 26 intervention beneficiaries, observations + 10 non-beneficiaries observations of the CHIEDZA sites + 7 study team observations.	Interviews with implementers: providers' experiences of reopening CHIEDZA, including their perceptions of how the intervention had changed in response to the restrictions and any impact this had. Interviews with beneficiaries: clients' experience of the adapted CHIEDZA intervention, and how these influenced their interactions with the intervention. Non-beneficiaries observations of the CHIEDZA sites: adaptations to CHIEDZA that had been made in the context of the pandemic. Study team observations: internal processes of adaptations of the intervention, and understanding the study

									team and provider perceptions of the intervention as it adapted.
Mahey, 2020 [15]	India	- Maternal and Newborn<br>- Reproductive health	- Due to the lockdown, routine outpatient departments (OPD) were closed.<br>- Hospital services, including infrastructure and manpower, were diverted to manage the cases of COVID-19 along with emergency services.	Department of Obstetrics and Gynecology of a University Hospital	Faculty and residents agreed that the best way forward would be to reorganize the roster: teams comprising a consultant, senior and junior residents, fellows, and interns from each unit were designated for an entire week. They managed all patients in the screening area, regardless of the unit to which they belonged. To take care of positive cases of COVID-19, there was a dedicated team each week, drawn from each of the three units in rotation. This method enabled each team to be posted on clinical duties for 1 week, with 1 to 2 weeks off clinical work, working on teaching and other non-clinical duties or remaining on standby in case they were needed. The handover from one team to the next at the end of each week was done through telecommunication.	3 months	Mixed Methods	Data from the Department of Obstetrics and Gynaecology	The number of health workers contracting COVID-19 infection, the number of contacts in case a positive patient unexpectedly comes to the delivery room or ward; the need of quarantine leaves; and adverse event(s). Residents' perspective regarding the new emergency roster, impact on surgical skills, impact of "off-emergency duty" time, what was felt to be missing, and drawbacks of the roster.
Malkin (I), 2022 [16]	Zimbabwe	- Child and Adolescent<br>- Reproductive health	- Shortages of personal protective equipment and recurring health worker strikes throughout the year, exacerbated by a Malaria outbreak.<br>- Fear of transmission, lockdowns, curfews, movement restrictions, and lack of public transport led to people's reduced use of Family	9 provinces	The Mhuri/Imuli (name of the intervention) implemented a modified outreach model that involved stationing outreach staff at health facilities to offer FP services including implants, copper intrauterine devices, oral contraceptive pills, injectables, male condoms, and female condoms. To support this model, community health volunteers and FP service providers directed clients to facilities when possible, while raising awareness of FP.	8 months	Mixed Methods	Data from the 9 provinces	Number of clients served

			Planning (FP) services.						
Malkin (II), 2022 [16]	Kenya	- Child and Adolescent<br>- Reproductive health	- Concerns about seeking family planning (FP) facility-based services during the pandemic.<br>- Adolescents feeling comfortable accessing FP services from older community health volunteers (CHVs) who traditionally provide these services.	30 facilities	The project recruited youth CHVs who were closer in age to the clients they served. Youth CHVs were trained on FP and basic contraceptive method counseling and paired with existing CHVs who served as mentors. Youth CHVs provided counseling and distributed pills and condoms. In addition, they mobilized and/or led community dialogues and small group discussions with adolescents on topics including body literacy and fertility awareness, menstruation, preventing unintended pregnancy, healthy timing and spacing of pregnancy, nutrition, pregnancy, and parenting.	6 months	Mixed Methods	Data from the 30 facilities	Number of adolescents and youth receiving contraceptive services
Malkin (III), 2022 [16]	Nigeria	- Child and Adolescent<br>- Reproductive health	- Restrictions limited youth's ability to travel to facilities and access SRH services, being provided by ongoing projects.	5 states in Southern Nigeria	A360 project provides safe SRH information through the in-person Life, Love, and Health (LLH) classes and SRH services regularly. The LLH curriculum offered information to adolescent girls on vocational skills, love, relationships, and health to generate demand for contraceptive uptake. In response to COVID-19 movement restrictions, A360 began digitizing the LLH classes via WhatsApp to safely reach adolescent girls with relevant SRH information and linkages to products and services. The WhatsApp LLH classes were offered in a group chat format by geographic clusters and included the option of one-on-one counseling with a provider who could refer and link girls with SRH facilities. A360 launched a new contraceptive education Facebook campaign and developed new partnerships with content creation and technical support partners.<br><br>A360 fast-tracked "Big Sistas," a pilot community-based program to distribute self-injection of subcutaneous depot medroxyprogesterone acetate (DMPA-SC), was adapted to adhere to social-distancing restrictions. Big Sistas are experienced and knowledgeable peer mobilizers	11 months	Mixed Methods	Data from the 5 states	Contraceptive method uptake and number of participants reached through the Facebook campaign.<br><br>Big Sistas trained, the number of adolescents that initiated DMPA-SC, number of adolescents that were referred.

					who are satisfied DMPA-SC users based in the community and could relate to the experience of choosing a contraceptive method. Big Sistas were tasked with training, referring, supplying, and supporting other adolescents interested in DMPA-SC through one-on-one counseling, following social distancing protocols.				
Marchiori, 2020 [17]	Brazil	- Maternal and Newborn<br>- Child and Adolescent	- With the spread of the virus, the number of cases in pregnant women, women in labor, and women who have recently given birth became part of the risk group<br>- Breastfeeding has demanded differentiated attention.	5 public hospitals from the Human Milk Bank (HMB) coordination	The actions of HMB services maintenance strategies by digital means as a way to ensure social distancing and breastfeeding during the pandemic. The strategies included video referrals, WhatsApp messaging, text messaging, and Internet referrals through websites (e.g., YouTube, Facebook).	Not possible to determine	Qualitative	5 intervention implementers	Open questions regarding the strategies and measures adopted by the HMB to maintain breastfeeding continuity during the times of COVID-19 including: How is the action to promote breastfeeding being carried out in times of protective measures? How does HMB work on breastfeeding protection in social isolation? What measures has HMB implemented to offer or maintain breastfeeding support? What are the strategies to face the pandemic, maintain social distance, and breastfeed in this scenario?
Moulaei, 2021 [18]	Iran	- Maternal and Newborn	- In pregnant women with preeclampsia, their diagnosis could be worsen if they get COVID-19.	Hospitals and medical centers affiliated to Kerman University of Medical Sciences	Designed a proper mHealth application, a self-care application, facilitating self-care for pregnant women, who suffer from preeclampsia in the COVID-19 pandemic. The following domains were covered by the mHealth application: user's profile, lifestyle, prevention, and control, and application capabilities.	10 days	Mixed Methods	15 intervention beneficiaries	User satisfaction using a Questionnaire for User Interaction Satisfaction (QUIS).

Moyo, 2020 [19]	Zimbabwe	- Maternal and Newborn<br>- Reproductive health	- Need to adapt service due to lockdown restrictions	Not specified	The researchers provide free obstetrics and gynecology consultations via SMS and WhatsApp. The telephone numbers of the researchers (volunteers implementing the intervention) were advertised on electronic and print media. The patients would send messages via WhatsApp or SMS to the doctor's number and the doctor would respond in real time or delay texting. The doctors would then communicate their recommendations to the patient. If deemed an emergency, the researcher would make arrangements for the patient to be attended at the nearest hospital or facility.	3 weeks	Quantitative	67 intervention beneficiaries)	Effectiveness of telemedicine, and patient satisfaction regarding services provided.
Murthy, 2022 [20]	India	- Maternal and Newborn<br>- Vaccination	- Resistance of parents to vaccinate their children due to negative beliefs about vaccines, lack or distrust of adequate information, and negative propaganda.<br>- Shortage of health workers, lockdowns, and lack of transportation slowed down the utilization of most preventive services.<br>- Fear of COVID-19 infection, lack of social distancing, and inadequate infection control practices affected health-seeking behavior.	6 health care facilities	Phone-call counselling interventions to support immunization: The initial call was made seven to ten days after the due date for immunization. At the end of each call, families who had not vaccinated their baby or had missed the scheduled vaccine were counseled for immunization. During counseling, tele-trainers emphasized the role of vaccines in the baby's health, addressed any fears, myths, or doubts the family had, gave information about where they can vaccinate their baby, and, if asked, gave the contact details of the local worker. Families who had not vaccinated their baby or were unreachable during the initial call were followed-up on after seven days. If families did not vaccinate their baby at the first follow-up call, they were further counseled. The second follow-up call was conducted fifteen days after the initial call.	Not possible to determine	Quantitative	2097 intervention beneficieries	Immunization uptake, existing immunization coverage, and reasons for non-vaccination

Nur, 2020 [21]	Indonesia	- Maternal and Newborn<br>- Reproductive health	- Need to adapt antenatal care (ANC) service due to lockdown restrictions	11 villages within a Public Health Center area	Midwives and pregnant women were selected to be part of a program on e-ANC. They were given Android devices and taught how to use them. These devices allowed the midwives to provide ANC via online content. The e-ANC was made up of very simple content. These include, among others, a complete ANC, counseling, information on the need for high-risk early detection, hemoglobin (hb), and iron (Fe) tablets monitoring, information on the danger of pregnancy, healthy living behavior during the pandemic, prevention methods, and the dangers and impact of the COVID-19 pandemic.	3 months	Quantitative	30 intervention beneficiaries + 20 intervention implementers	Effect of COVID-19 periods on ANC coverage of pregnant women. The effects of ANC on the improved midwife participation in counseling, high-risk early detection, Hb monitoring, and the provision of Fe tablets. Effects of e-ANC on pregnant women for counseling, high-risk early detection, Hb examination, and provision of Fe tablets. Impact of disorders on women's reproductive health.
Ozalp Akin, 2022 [22]	Turkey	- General health service<br>- Child and Adolescent	- Social distancing restrictions imposed barriers to children accessing monitoring development programs.<br>- Caregiver social isolation, stress, burden, and burnout, compromising the mental health and well-being of children and families.	University Developmental Pediatrics Division clinic	The Guide for Monitoring Children Development (GMCD) intervention was adapted to be delivered using telehealth. The clinician delivered the GMCD intervention during a single 40-minute telephone call. Information on all of the developmental domains, strengths, delays in development, and psychosocial strengths and risk factors were identified. R Recommendations on how to support the child's development based on the child's functioning and strengths were discussed. Further, a mutually developed plan was made with the caregiver on how to address risk factors and needs, employing a strengths-based approach. For cases in which the clinicians or caregivers had doubts regarding the child's functioning or how to promote the child's development, clinicians asked the caregivers to record short relevant videos and to send these back via WhatsApp.	3 months	Mixed Methods	114 intervention beneficiaries	GMCD Applicability Questionnaire evaluating comprehensibility, partnership, perceived effectiveness, adaptability, and satisfaction. Also, feedback from the caregivers regarding the intervention. GMCD Video Observation Tool assessed if the following aspects were or not observed: developmental functioning of the child in the GMCD domains, activities and participation of the child, and whether the caregivers' were engaged in promoting the child's development.
Phiri, 2022 [23]	Zambia	- Child and Adolescent<br>- Reproductive health	- Adolescents and young people are at increased risk of HIV and other sexually transmitted infections (STIs).	2 communities	Yathu Yathu is an intervention created before COVID-19 to give sexual and reproductive health services to adolescents and young adults. The intervention included spaces (hubs) where the following services were offered: HTS, STI screening, condom distribution, and information and provision of contraceptives. Information and education on SRH is provided through edutainment and CSE sessions. Social media platforms, including Facebook,	9 months	Mixed Methods	Data routinely collected in the 2 communities.	The total number of followers and number of interactions with posts on Facebook. Observations of service delivery interviews with young people accessing services at the hubs and focus group discussions to assess

			- There is a need for information/ education on STIs/HIV and access to diagnostic services, including HIV testing services (HTS).<br>- The pandemic aggravated the problem of access to these types of services.		and WhatsApp groups, were launched to provide staff a communication route with participants. Adaptations due to COVID-19 included the provision of CSE sessions via Facebook and WhatsApp in which COVID-19 information videos were promoted. Other adaptations included: infection control, standard operating procedures to guide service delivery, staff provided with personal protective equipment, all hubs required to have a “reception” desk outside where participants were provided information about COVID-19, a contact tracing register was completed, and a fabric mask provided to all participants before entry, among others.				experiences and perceptions of service delivery. WhatsApp reports on the number of participants, topics discussed, and questions raised were submitted per community.
Richardson, 2020 [24]	Nigeria	- Child and Adolescent	- Seasonal Malaria chemoprevention (SMC) campaign need to adapt SMC delivery to ensure the safety of distributors, beneficiaries and communities.<br>- Misinformation regarding COVID-19 about its prevention, cure and infection among caregivers of children eligible for SMC.	6 states	The Malaria Consortium created a training flipbook for the SMC distributors to guide the administration of the programme and also distribute health messages regarding the prevention of COVID-19 to children caregivers.	4 months	Quantitative	40,157 intervention implementers	Effectiveness of the public health campaign including knowledge of COVID-19 prevention, knowledge of symptoms, and belief in COVID-19 misinformation
Severini, 2022 [25]	Brazil	- General health<br>- Child and Adolescent	- Need to adapt service due to restrictions.<br>- The leave of numerous health professionals	1 public tertiary hospital	Consultations were performed by video call rather than in person in a pediatric center. The healthcare staff was trained to deliver this type of service. Parents were given instructions on how to access the service by email and social networks and at the end they were given a satisfaction survey.	3 months	Quantitative	Data from 255 consultations of 140 intervention beneficiaries.	Satisfaction score, the number of consultations, wait-up timing, duration of the consultation, reported difficulties in access to

			due to the pandemic.						telehealth, and number of referrals.
Sulaman, 2022 [26]	Pakistan	- Maternal and Newborn	- Need to adapt ante-natal medical service due to Covid lockdown restrictions	1 hospital	The Shifa International Hospital implemented a telemedicine program to attend routine antenatal care. The whole process from making the appointment to having the consultation was performed by telemedicine.	16 months	Quantitative	132 intervention beneficiaries	- Number of appointments, staff attitude, medical consultation, communication, and future use of telemedicine.
Van der Linde, 2021 [27]	South Africa	- Maternal and Newborn<br>- Child and Adolescent	- The already limited access to early and continuous developmental care for children was exacerbated due to COVID-19 restrictions.<br>- Low availability of resources due to socio-economic disparities.	1 department from a university	Different types of tele-intervention sessions were provided by several caregivers as part of early intervention services offered to the public by the Department of Speech-Language Pathology and Audiology at the University of Pretoria when lockdown restrictions prevented in-person sessions.	5 months	Qualitative	30 caregivers and student clinicians	Stakeholders' (caregivers') perspectives of telehealth across the following themes: practicality, convenience and resource-saving, safety amid a pandemic, the tele-assessment format used, and caregiver knowledge.
Vetcho, 2022 [28]	Thailand	- Maternal and Newborn	- Implementation of Family Centered Care (FCC) in low-resource health settings is challenging to implement.<br>- COVID-19 has impacted care delivery for neonates and their families in the neonatal intensive care units (NICUs); visitation	NICU from 1 hospital	The FCC innovations flowchart provided: 1) Flexible visitation to daily updates (flexible hour) 1 h/day (over a flexible period between 10 AM–4 PM. (6 h) and restricted visitors to only parents, excluded during procedures and resuscitation. 2) Telephone call (at least three times per week) to update newborn progress and treatment in NICU. 3) Interdisciplinary family meetings for complex care situations. 4) Information booklet, e-booklet, and paper-based (revised) with the details for the COVID-19 situation including NICU introduction: environment, staff, NICU policies, visiting management, important information, and parental education during admission to NICU.	2 months	Quantitative	83 pairs of parents (i.e., mother and father of neonate participated) (35 pre; 48 posts), which represented 102 neonates. For the NICU healthcare team, 20 participated).	The Perceptions of FCC–Parent and Perceptions of FCC–Staff instruments assessing the domains of respect, collaboration, and support. Respect refers to recognizing the family's rights in the hospital. Collaboration reflects the partnership role of parents in caring for their neonate. Support focused on staff demonstrating support for the families' needs during the neonates' hospitalization.

			policies, developmental care, and communication practices.						
Wahyul, 2021 [29]	Indonesia	- Mental health<br>- Maternal and Newborn Health<br>- Reproductive health	- Need to adapt service antenatal care service due to lockdown restrictions and closures of facilities.	1 public health center	Recent graduates from a midwifery school conducted telehealth monitoring and counselling on a volunteer basis. Assistance was carried out from early pregnancy until postpartum is complete. The information is given via Whatsapp, telephone, and video call.	Not specified	Quantitative	30 intervention beneficiaries + 27 no-intervention beneficiaries	Knowledge on pregnancy, childbirth and post-partum, and knowledge on mental health measured as depression risk of postpartum mothers.
Winter, 2022 [30]	Zambia	- Child and adolescent<br>- Vaccination	- Disruptions in the administration of routine vaccination.	National level	Zambia's Child Health Weeks took place despite the pandemic. These vaccination activities included COVID-19 precautionary protocols including the use of personal protective equipment and minimum distance requirements for individuals seeking care.	2 weeks	Quantitative	National level	Number of vaccinated children among districts, coverage, and susceptibility of the population

References

1 Ahmad B. Infant and young child feeding in emergencies: programming adaptation in the context of COVID-19 in Lebanon. F Exch - Emerg Nutr Netw ENN. 2021;57–9.

2 Ahmed S, Hussein B, Barasa E. Adapting infant and young child feeding interventions in the context of COVID-19 in Somalia. F Exch - Emerg Nutr Netw ENN. 2021;54–6.

3 Barua M, Saha A, Chowdhury S, Sajow S. Implementation of a community-based referral project to improve access to emergency obstetric and newborn care in Rohingya population during COVID-19 pandemic in Bangladesh. BMJ Innov. 2022;8:247–54.

4 Devi R, Parvathi T, Murugan R, Sagili H, Lakshminarayanan S. Outcomes of teleconsultation services and patient satisfaction among pregnant women delivering at a tertiary care center in South India during coronavirus disease 2019 pandemic. Indian J Public Health. 2022;66:210–3.

5 Doubova S, Arsenault C, Contreras-Sanchez S, Borrayo-Sanchez G. The road to recovery: an interrupted time series analysis of policy intervention to restore essential health services in Mexico during the COVID-19 pandemic. J Glob Health. 2022;12:05033.

6 Enyama D, Chelo D, Noukeu Njinkui D, Mayouego Kouam J, Fokam Djike Puepi Y, Mekone Nkwele I, et al. Impact of the COVID-19 pandemic on pediatricians' clinical activity in Cameroon. Arch Pediatr. 2020;27:423–7.

7 Gadappa S, Badgire S, Deshpande S. Implementation Of Quality Improvement Inbirth Companion Policy At Government Medical College And Hospital in the "NEW NORMAL" of COVID 19. Eur J Mol Clin Med. 2022;9:739–49.

8 Goyal L, Garg P, Verma M, Kaur N, Bakshi D. Effect of restrictions imposed due to COVID-19 pandemic on the antenatal care and pregnancy outcomes: A prospective observational study from rural North India. BMJ Open. 2022;12:e059701.

9 Hensen B, Gondwe M, Phiri M, Schaap A, Simuyaba M, Floyd S, et al. Access to menstrual hygiene products through incentivised, community-based, peer-led sexual and reproductive health services before and during the COVID-19 pandemic: findings from the Yathu Yathu trial. BMC Public Health. 2022;22:554.

10 Joshi N, Bakshi H, Chatterjee A. Initiative to improve quality of paediatric ward-round documentation by application of "SOAP" format. BMJ open Qual. 2022;11:e001472.

11 Khader Y, Maalouf W, Khdair M, Al-Nsour M, Aga E, Khalifa A, et al. Scaling the Children Immunization App (CIMA) to Support Child Refugees and Parents in the Time of the COVID-19 Pandemic: A Social Capital Approach to Scale a Smartphone Application in Zaatari Camp, Jordan. J Epidemiol Glob Health. 2022;12:7–12.

12 Juan L, YongChun L. Intervention effect of the video health education model based on solution-focused theory on adolescents' mental health during the COVID-19 pandemic. Iran J Public Health. 2021;50:2202–10.

13 Li L, Li Z, Wan W, Li J, Zhang Y, Wang C, et al. Management of Follow-Up With Preterm Infants During the Outbreak in China. Front Pediatr. 2021;9:637275.

14 Mackworth-Young C, Mavodza C, Nyamwanza R, Tshuma M, Nzombe P, Dziva Chikwari C, et al. "Other risks don't stop": adapting a youth sexual and reproductive health intervention in Zimbabwe during COVID-19. Sex Reprod Heal Matters. 2022;30:2029338.

15 Mahey R, Sharma A, Kumari A, Kachhawa G, Gupta M, Meena J. The impact of a segregated team roster on obstetric and gynecology services in response to the COVID-19 pandemic in a tertiary care center in India. Int J Gynecol Obstet. 2020;151:341–6.

16 M., Malkin M, A.K., Mickler A, Ajibade T, Coppola A, Demise E, Derera E, et al. Adapting High Impact Practices in Family Planning During the COVID-19 Pandemic: Experiences From Kenya, Nigeria, and Zimbabwe. Glob Heal Sci Pract. 2022;10:e2200064.

17 Marchiori G, Alves V, Pereira A, Vieira B, Rodrigues D, Dulfe P. Nursing actions in human milk banks in times of COVID-19. Rev Bras Enferm. 2020;73:e20200381.

18 Moulaei K, Bahaadinbeigy K, Ghaffaripour Z. The design and evaluation of a mobile based application to facilitate self-care for pregnant women with preeclampsia during covid-19 prevalence. J Biomed Phys Eng. 2021;11:551–60.

19 Moyo J, Madziyire G. Use of telemedicine in obstetrics and gynaecology in zimbabwe during a lockdown period. Pan Afr Med J. 2020;35:89.

20 Murthy S, Sawant M, Doreswamy S, Pothula S, Yan S, Pathani T, et al. Supporting Immunization Uptake during a Pandemic, Using Remote Phone Call Intervention among Babies Discharged from a Special Neonatal Care Unit (SNCU) in South India. Vaccines. 2022;10:507.

21 Nur R, Radiah S, Aulia U, Dwilarasati R, Patui N, Mantao E, et al. Effects of electronic technology antenatal care (E-anc) on midwives and pregnant women during the covid-19 period. Open Access Maced J Med Sci. 2020;8:115–21.

22 Ozalp Akin E, Akbas A, Atasoy S, Kanatli M, Ince Acici S, Mustafayev R, et al. Applicability of the Guide for Monitoring Child Development as a Telehealth Delivered Intervention During the Pandemic. Front Pediatr. 2022;10:884779.

23 Phiri M, Hensen B, Schaap A, Sigande L, Simuyaba M, Simwinga M, et al. Adapting community-based sexual and reproductive health services for adolescents and young people aged 15-24 years in response to COVID-19 in Lusaka, Zambia: the implications on the uptake of HIV testing services. BMC Health Serv Res. 2022;22:503.

24 S., Richardson S, Ibinaiye T, Nikau J, Oresanya O, Marasciulo M, Roca-Feltrer A, et al. COVID-19 knowledge, beliefs, prevention behaviours and misinformation in the context of an adapted seasonal malaria chemoprevention campaign in six northern Nigerian States. Trop Med Health. 2020;48:101.

25 Severini R, Oliveira P, Couto T, Simon Junior H, Andrade A, Nanbu D, et al. Fast, cheap and feasible: implementation of pediatric telemedicine in a public hospital during the COVID-19 pandemic. J Pediatr (Rio J). 2022;98:183–9.

26 Sulaman H, Akhtar T, Naeem H, Saeed G. Beyond COVID-19: Prospect of telemedicine for obstetrics patients in Pakistan. Int J Med Inform. 2021;158:104653.

27 Linde J van der, Eccles R, Toit M du. Supporting early childhood development during COVID-19 using telehealth: stakeholders' perspectives. South African Heal Rev. 2021;255–62.

28 Vetcho S, Cooke M, Petsky H, Saito A, Ullman AJ. Family-centred care change during COVID-19. Nurs Crit Care. 2022;27:460–8.

29 Wahyul A. The effects of telehealth during pregnancy on maternal knowledge and postpartum mental health in the covid-19 pandemic. Indian J Forensic Med Toxicol. 2021;15:2834–41.

30 Winter A, Takahashi S, Carcelen A, Hayford K, Mutale W, Mwansa F, et al. An evaluation of the early impact of the COVID-19 pandemic on Zambia's routine immunization program. medRxiv (pre-print). 2022.
